# Self-esteem assessment of young female university students according to race/skin color criteria*

**DOI:** 10.1590/1518-8345.3866.3362

**Published:** 2020-10-19

**Authors:** Monalisa Nanaina da Silva, Juliana Cristina dos Santos Monteiro

**Affiliations:** 1 Universidade de São Paulo, Escola de Enfermagem de Ribeirão Preto, PAHO/WHO Collaborating Centre for Nursing Research Development, Ribeirão Preto, SP, Brazil.

**Keywords:** Black Population, Self Esteem, Mental Health, Women’s Health, Youth, Student Health, População Negra, Autoestima, Saúde Mental, Saúde da Mulher, Juventude, Saúde do Estudante, Población Negra, Autoestima, Salud Mental, Salud de la Mujer, Juventud, Salud del Estudiante

## Abstract

**Objective:**

to evaluate and compare the self-esteem of young female university students aged between 18 and 24 years old according to race/skin color criteria.

**Method:**

a cross-sectional and quantitative study, developed with 240 undergraduate female students from a public Brazilian university. Data collection took place online through a structured questionnaire that included the participants’ sociodemographic and lifestyle habits, and the Rosenberg Self-Esteem Scale. For data analysis, descriptive statistics, association test, and comparison of means were used.

**Results:**

most of the young women had a mean level of self-esteem. No statistically significant association was found among the “self-esteem level” and “self-reported skin color or race” variables.

**Conclusion:**

although no significant association was identified between self-reported skin color or race and level of self-esteem, young black women have lower mean self-esteem scores than young non-black women. Strategies that strengthen the self-esteem of young female university students are necessary to prevent harms to their physical and mental health, and, consequently, to their academic performance.

## Abstract (pt)

**Objetivo:**

avaliar e comparar a autoestima de jovens universitárias com idade entre 18 e 24 anos segundo critério raça/cor.

**Método:**

estudo transversal, quantitativo, desenvolvido com 240 graduandas de uma universidade pública brasileira. A coleta de dados ocorreu de modo online através de um questionário estruturado que contemplava as características sociodemográficas e de hábitos de vida das participantes, e a Escala de Autoestima de Rosenberg. Para análise dos dados utilizou-se estatística descritiva, teste de associação e comparação de médias.

**Resultados:**

a maioria das jovens apresentou nível médio de autoestima. Não foi encontrada associação estatisticamente significativa entre as variáveis “nível de autoestima” e “cor ou raça autorreferida”.

**Conclusão:**

embora não tenha sido identificada associação significativa entre cor ou raça autorreferida e nível de autoestima, jovens negras apresentam escores médios de autoestima inferiores ao de jovens não negras. Estratégias que fortaleçam a autoestima das jovens universitárias são necessárias para prevenir prejuízos à sua saúde física, mental, e, consequentemente, ao seu desempenho acadêmico.

## Abstract (es)

**Objetivo:**

evaluar y comparar la autoestima de jóvenes universitarias de entre 18 y 24 años en función de los criterios de raza/etnia.

**Método:**

estudio transversal, cuantitativo, desarrollado con 240 estudiantes universitarias de una universidad pública brasileña. La recopilación de datos se realizó *online* a través de un cuestionario estructurado que incluía los hábitos sociodemográficos y de estilo de vida de las participantes, y la Escala de Autoestima de Rosenberg. Para el análisis de datos, se utilizó estadística descriptiva, prueba de asociación y comparación de medias.

**Resultados:**

la mayoría de las mujeres jóvenes presentaban un nivel de autoestima promedio. No se encontró asociación estadísticamente significativa entre las variables “nivel de autoestima” y “etnia o raza autodeclarada”.

**Conclusión:**

aunque no se identificó una asociación significativa entre la etnia o raza autodeclarada y el nivel de autoestima, las jóvenes negras presentan puntajes promedio de autoestima más bajos que las jóvenes no negras. Es necesario implementar estrategias que fortalezcan la autoestima de las jóvenes universitarias para evitar daños a su salud física, mental y, en consecuencia, a su rendimiento académico

## Introduction

Self-esteem is defined as the judgment that the individuals make and keep about themselves, it is built up from the value that they give to themselves, generating a feeling of appreciation or disgust that defines the subjects’ self-perception^(^ 1 ^)^.

Individuals may have a low, medium or high level of self-esteem^(^ 1 ^)^. High self-esteem is manifested by the acceptance of responsibility for their own actions, by the ability to take reasonable risks, assuming total command and control over their own lives, including the adoption of healthy behaviors^(^ 2 ^)^. Low self-esteem individuals focus on trying to prove themselves to others. They generally lack confidence in themselves, and often doubt their own value and acceptability. People with medium self-esteem experience fluctuations in relation to self-concept, alternating between feelings of self-approval and self-rejection^(^ 1 ^-^ 2 ^)^.

Self-esteem is an important indicator of mental health^(^ 3 ^)^. High self-esteem scores have been associated with positive health-related practices^(^ 4 ^)^, while low self-esteem seems to be closer to risk behaviors, such as suicidal behavior^(^ 5 ^)^.

Scientific evidence has shown high engagement of the young population in health risk behaviors, such as physical inactivity, unhealthy eating habits, alcohol and other drug abuse, and risky sexual behaviors^(^ 5 ^-^ 7 ^)^. Within this context, strengthening young people’s self-esteem has shown to be a promising way to prevent these behaviors^(^ 4 ^,^ 6 ^)^; therefore, scientific interest is focused on assessing the level of self-esteem of this population today.

Regarding gender differences, due to conflicts with self-image standards, women may have lower rates of self-esteem^(^ 8 ^)^. One of the few national studies on this subject involving 1,151 elementary, high school, and higher education students, aged between 10 and 30 years, identified differences between genders only within the age group of 16 to 19 years old, where the mean of the men in this group was significantly higher than that of the women^(^ 9 ^)^. Still, the researchers found significant differences between the self-esteem of elementary and high school students and higher education students, suggesting that university life has been experienced as a stressful phase for the young individuals^(^ 9 ^-^ 10 ^)^.

Regarding the racial differences in self-esteem, researchers point out the link between racial discrimination and traumatic symptoms in university students^(^ 11 ^-^ 13 ^)^. Although it is assumed that the self-esteem of black women is more compromised when compared to non-black women, given the multiple violence to which they are exposed due to racism^(^ 11 ^-^ 12 ^)^, no studies were found that were dedicated to comparing the levels of self-esteem among these populations to support such an assumption in the Brazilian context. On the other hand, international studies comparing the effects of ethnic-racial identity on self-esteem show that there is a higher level of self-esteem among young black women than in any other young female ethnic group^(^ 14 ^)^.

In view of the above, considering the social relevance of the theme for the mental health of young people from public universities, and due to the scarcity of national studies that assess the self-esteem of the university population based on racial identities, it was decided to carry out this study with the objective of evaluating and comparing the self-esteem of young female university students according to race/skin color criteria.

## Method

This is a quantitative and cross-sectional study developed in a public university campus located in the inland of the state of São Paulo. This campus has courses distributed in the Exact, Health, Human, and Biological science areas.

Based on previous studies that demonstrate an association between exposure to racial discrimination and negative mental health outcomes^(^ 11 ^-^ 13 ^)^ the hypothesis was set out that young black female university students have a lower level of self-esteem compared to young non-black women.

The study population consisted of all the young women students from the first and last years of all the face-to-face undergraduate courses in the studied campus, aged between 18 and 24 years old. According to the information made available by the university’s Information Service, in 2017, 752 women enrolled in undergraduate courses on that campus, and 668 students were trained (personal information), that is, the population of interest in this study was equivalent to approximately 1420 women (freshmen and trainees). The exclusion criteria were the following: young individuals who identified with the male gender, young people who did not fit into the freshman or graduating categories, young people under 18 years old or 25 years old or more at the time of collection. The exclusion criterion considering this age limit is justified because, in this study, the classification used by the National Youth Policy was followed, which divides subjects aged between 15 and 29 years old into three groups: young people aged 15 to 17 years old, referred to as young-adolescents; young people aged 18 to 24 years old, as young-young people; and young people aged 25 to 29 years old, as young-adults^(^ 15 ^)^.

Two instruments were used for data collection. The first was a structured questionnaire that included the identification data, the sociodemographic characteristics and life habits of the participants (age, race/self-reported skin color, course, year of the course, occupation, religion, smoking, drinking, use of illicit drugs, marital status, type of school attended). This instrument was developed and based on the scientific literature and on previous research studies carried out in the study area. The second instrument used was the Rosenberg Self-Esteem Scale. This is a one-dimensional measure of self-esteem, consisting of ten statements related to a set of positive and negative feelings of self-acceptance to assess global self-esteem, which is classified as low (10 to 20 points), mean (20 to 30 points) and high (30 to 40 points)^(^ 9 ^,^ 16 ^)^. Items are answered on a Likert-type scale in which the answer options are the following: totally agree, agree, disagree, and totally disagree. Each of these options is assigned a number ranging from 1 to 4 points. In Brazil, this instrument was adapted and validated for research and is currently in the public domain, easy and quick to apply^(^ 9 ^)^.

The instruments were transferred to the Research Electronic Data Capture (REDCap) electronic data capture tool^(^ 17 ^)^. The REDCap software is a web-based platform that aims to simplify and streamline the development of electronic forms of data capture to be used in research studies^(^ 17 ^)^. Data collection took place between May and September 2018. All the students were invited to take part in the survey through electronic mail. On the online survey page, after being aware of the research and the ethical aspects, those who agreed to participate expressed their agreement electronically by clicking on the participation acceptance button on the page containing the Free and Informed Consent Form (FICF).

According to the records made available by REDCap, there were 1,025 accesses to the data collection instrument between the months it was available to students. However, only 540 respondents completed it properly. Of these, 240 met the inclusion criteria proposed by the researchers.

Data was exported from REDCap by automatic procedures that are specific to the platform, directly to a spreadsheet in Microsoft Excel. All the data were analyzed using the Statistical Analysis System SAS^®^ 9.4 statistical program. The participants were characterized based on descriptive statistics. For the analyses, the data referring to the “self-reported race/skin color” variable were categorized into two subgroups: “young black women” (participants who declare themselves black- and brown-skinned), and “young non-black women” (participants who declare themselves white- and yellow-skinned). This categorization is in accordance with the race/skin color classification system of the Brazilian Institute of Geography and Statistics (*Instituto Brasileiro de Geografia e Estatistica*, IBGE)^(^ 18 ^)^. In the classification system in question, five categories of skin color or ethnicity are used: white, black, brown, yellow, and indigenous, and the black Brazilian population is constituted from the aggregation of self-declared black- and brown-skinned subjects^(^ 18 ^)^.

Fisher’s exact test was used to verify the association between the qualitative variables. To compare the means of the self-esteem scores between black and non-black young women, the T-Student Test was used. For all the statistical analyses, a significance level of 5% (α=0.05) was considered.

This study was submitted to and approved by the Research Ethics Committee (*Comitê de* Ética *em Pesquisa*, CEP) of the Ribeirão Preto Nursing School at the University of São Paulo, under protocol No. 80315217.2.0000.5393.

## Results

240 young female university students took part in this study, with a mean age being 21 years old, predominantly white-skinned, attending the last year of graduation, who did not perform paid work and did not actively participate in any religion. Most of them had a partner but did not live together, and studied entirely in a private school before entering higher education.

Regarding life habits, the largest proportion of young women did not smoke and did not use illicit drugs. It is noteworthy that 86.6% of the participants drank alcoholic beverages, with 34.1% declaring their use once or twice a week.

Table 1 shows the distribution of the study participants according to sociodemographic characteristics and lifestyle.

**Table 1:** Distribution of the study participants according to self-reported race/skin color, year of the course, occupation, religion, marital status, type of school attended, smoking, alcohol consumption, and use of illicit drugs. Ribeirão Preto, SP, Brazil, 2018

Variables	Frequency	%
Self-reported race/skin color (n=240)		
White	180	75.0
Black	14	5.8
Brown	34	14.1
Asian	12	5.0
Course year (n=240)		
I entered university in 2018	108	45.0
My graduation forecast is for 2018	132	55.0
Do you have paid work? (n=240)		
Yes	39	16.2
No	201	83.7
Do you participate actively in some religion (n=240)		
Yes	47	19.5
No	193	80.4
Marital Status (n=240)		
Single	111	46.2
I have a partner, but we don't live together	115	47.9
Married or living together with a partner	14	5.8
Regarding your years of study, to which one of the following groups do you belong? (n=240)		
I studied entirely in a private school	89	37.0
I studied most of the time in a private school	49	20.4
I studied entirely in a public school	69	28.7
I studied most of the time in a public school	33	13.7
Smoking (n=240)		
Yes	19	7.9
No	221	92.0
Habitual consumption of alcoholic beverages (n=240)		
Drinking every or almost every day	1	0.4
Drinking once or twice a week	82	34.1
Drinking once or twice a month	81	33.7
Drinking less than once a month	44	18.3
Not drinking	32	13.3
Use of illicit drugs (n=240)		
Using every or almost every day	5	2.0
Using once or twice/week	9	3.7
Using one to three times/month	16	6.6
Using less than once/month	41	17.0
Not using	169	70.4
Total	240	100

Regarding the self-esteem assessment, the distribution of the results obtained through the application of the Rosenberg Self-Esteem Scale is shown in Table 2. It is observed that the largest proportion of participants (53.7%) had a medium level of self-esteem. The mean score was 27.7 points with a standard deviation of 5.9, a minimum score of 11 points, and a maximum of 40 points.

**Table 2:** Distribution of the study participants according to their level of self-esteem. Ribeirão Preto, SP, Brazil, 2018

Variable	Frequency	%
Self-esteem (n=240)		
Low (11-20)	28	11.6
Medium (21-30)	123	53.7
High (31-40)	33	34.5
Total	240	100

The result of the association test between self-esteem level and self-reported race/skin color is shown in Table 3. It is observed that there was no statistically significant association between the variables analyzed.

**Table 3:** Association between level of self-esteem according to race/skin color criteria. Ribeirão Preto, SP, Brazil, 2018

	Self-esteem level				
Self-reported race/skin color	Low n (%)	Medium n (%)	High n (%)	Total n (%)	*p*-value*
					
Black (black- and brown-skinned)	8 (28.5)	29 (22.4)	11 (13.2)	48 (20.0)	0.1136
Not black (white and yellow-skinned)	20 (71.4)	100 (77.5)	72 (86.7)	192 (80.0)	
Total	28 (11.6)	129 (53.7)	83 (34.5)	240 (100.0)	

* Fisher's Exact Test

Table 4 shows the comparison between the means of the self-esteem scores of the study participants according to the race/skin color criterion. Although no statistical significance was found (p = 0.8239), it is observed that young non-black women had a higher self-esteem mean level than young black women.

**Table 4:** Comparison of the means of the self-esteem scores of the study's participants according to race/skin color criteria. Ribeirão Preto, SP, Brazil, 2018

		Self-esteem level		
Self-reported race/skin color	n	Mean	Standard Deviation	*p*-value*
Black (black- and brown-skinned)	48	26.3	5.9	0.8239
Non-black (white- and yellow-skinned)	192	28.1	5.8	

* t-Student test = (t=1.88)

## Discussion

The sociodemographic characteristics of age, paid work, religion, and type of school attended in high school identified in the participants of this study are similar to those of participants in previous studies^(^ 19 ^-^ 20 ^)^.

Regarding the self-reported race/skin color, most of the participants declared themselves white-skinned, as well as in a research study^(^ 19 ^)^ which identified, over a period of 10 years, a mean enrollment of self-declared white-skinned students of 76.4%, while that of self-declared black-skinned students (black- and brown-skinned) corresponded to 21.7% (5.4% and 16.2%, respectively), evidencing that, although a growing entrance of the black Brazilian population in universities can already be observed, in some of them, this space continues to be mainly occupied by white-skinned individuals^(^ 21 ^-^ 22 ^)^.

With regard to lifestyle characteristics, most of the participants denied the habit of smoking and consuming illicit drugs, while 34.1% reported drinking alcoholic beverages once or twice a week. These findings have also been observed in other national and international studies and have attracted attention, given the negative consequences that the consumption of alcohol and other drugs can trigger on the health of these young women^(^ 23 ^-^ 24 ^)^.

Most of the participants in this study had a medium level of self-esteem, which indicates that young women experience fluctuations in self-esteem, sometimes self-worth and sometimes self-discarding^(^ 1 ^)^. Previous studies also developed with the university population corroborate our findings^(^ 25 ^-^ 27 ^)^.

A number of authors agree that, as well as other non-cognitive skills, self-esteem is important in determining health choices among young people, allowing them to assess, based on the value they attribute to themselves, which behaviors they will or will not adopt^(^ 4 ^,^ 6 ^)^. Regarding the university population, a study identified that low self-esteem predicted more symptoms of depression and anxiety among the students^(^ 28 ^)^. Healthy self-esteem, in turn, made it possible to better face the stressors recognized within the academic life. Young people with low self-esteem are more likely to get involved in risky behaviors, such as substance use and unprotected sex, as strategies for coping with their problems^(^ 28 ^)^.

The results showed that there was no statistically significant association between the “level of self-esteem” and “self-reported race/skin color” variables, thus rejecting the pre-established hypothesis. However, the observation of the participants’ self-esteem means indicates that young black women have lower mean scores than those of non-black participants.

Although there is logic in the assumption that groups which face disadvantages and social discrimination have low self-esteem when compared to those that do not experience these disadvantages, scientific findings have revealed antagonistic results to these assumptions, especially regarding the black female population^(^ 14 ^,^ 29 ^)^. One of the justifications offered to explain the difference in self-esteem between black women and women from other racial groups is the strength of racial identity^(^ 11 ^)^, which is understood as the feeling of belonging and attachment to their racial identity^(^ 30 ^)^. Having a strong racial identity can allow young women, when identifying discriminatory experiences, to perceive discrimination as an oppressive mechanism that starts from society towards them, and not the other way around, that is, the young women who have a strengthened racial identity externalize the experienced racism, instead of internalizing it and taking the blame for the context of discrimination that they experience^(^ 11 ^)^.

Scientific evidence from American studies shows that black women who experience racism and sexism in their daily lives suffer psychically, presenting symptoms of trauma^(^ 11 ^,^ 31 ^-^ 32 ^)^. However, the strength of their racial identity works to protect these women against internalization and blame for the transgressions experienced throughout life. Therefore, the multiple forms of oppression have little effect on their self-esteem, if they present stable racial identities^(^ 11 ^)^.

In the Brazilian context, however, this culture of strengthening racial identity is still little stimulated and, although it is already possible to observe the reflexes of years of articulation of the Brazilian black movement in the struggle to re-signify the identity of the black person^(^ 33 ^)^, the sense of racial collectivity is not so natural. Thus, young black Brazilian women may not yet have a strengthened sense of racial identity, allowing racism and sexism to have deleterious effects on their self-esteem^(^ 13 ^,^ 34 ^)^, which in turn affects their physical and mental health.

The findings of this study are justified by being conducted in a public university, a place that, like other prestigious spaces, has been historically denied to the black population^(^ 34 ^)^. Thus, even though it is a setting full of challenging situations, among them the setbacks of staying for years in an extremely elite and racist space^(^ 34 ^)^, occupying the university space reflects overcoming, achievement, and an opportunity for the expansion of knowledge for black women, factors that echo positively in their self-esteem^(^ 35 ^-^ 37 ^)^. Therefore, it is possible that the university status contributed to the self-assessment of young black women in this study, placing their self-esteem close to that of non-black young women.

The research has some limitations, such as having been developed in only one higher education institution. The sample size is also a limitation when considering the total number of undergraduate students at the studied institution. The contribution of the study to the advancement of scientific knowledge is in the new reflections on health promotion from the strengthening of the self-esteem of young female university students. This study is a pioneer in assessing the self-esteem of young female university students based on their racial identities and draws the attention to the need to produce scientific data that characterize the Brazilian black population, as well as the importance of analyses that consider the race/skin color item.

## Conclusion

Most of the young women had a medium level of self-esteem. The hypothesis that young black female university students would show a lower level of self-esteem when compared to non-black female university students was tested, with this hypothesis being rejected by the researchers, since there was no statistically significant difference.

Considering that medium levels of self-esteem reflect fluctuations in the self-concept, and that moments of self-devaluation predispose to involvement in risky health behaviors, strategies are needed to strengthen the self-esteem of young female university students in order to avoid harms to their physical and mental health and, consequently, to their academic performance.

New analyses are necessary to contribute to the understanding of the factors that influence the self-esteem of the young university population.
